# Cyclophilin A: a key player for human disease

**DOI:** 10.1038/cddis.2013.410

**Published:** 2013-10-31

**Authors:** P Nigro, G Pompilio, M C Capogrossi

**Affiliations:** 1 Centro Cardiologico Monzino-IRCCS, Laboratorio di Biologia Vascolare e Medicina Rigenerativa, Milan, Italy; 2 Laboratorio di Patologia Vascolare, Istituto Dermopatico dell'Immacolata-IRCCS, Rome, Italy

**Keywords:** CyPA, cardiovascular diseases, viral infections, neurodegeneration, cancer, rheumatoid arthritis

## Abstract

Cyclophilin A (CyPA) is a ubiquitously distributed protein belonging to the immunophilin family. CyPA has peptidyl prolyl *cis*-*trans* isomerase (PPIase) activity, which regulates protein folding and trafficking. Although CyPA was initially believed to function primarily as an intracellular protein, recent studies have revealed that it can be secreted by cells in response to inflammatory stimuli. Current research in animal models and humans has provided compelling evidences supporting the critical function of CyPA in several human diseases. This review discusses recently available data about CyPA in cardiovascular diseases, viral infections, neurodegeneration, cancer, rheumatoid arthritis, sepsis, asthma, periodontitis and aging. It is believed that further elucidations of the role of CyPA will provide a better understanding of the molecular mechanisms underlying these diseases and will help develop novel pharmacological therapies.

## Facts

- CyPA is a critical mediator for cardiovascular diseases.
- High CyPA expression correlates with poor outcome of patients with inflammatory diseases.
- CyPA regulates the infection and replication of several viruses affecting humans.
- CyPA is generally overexpressed in cancer and regulates malignant transformation and metastasis.
- CyPA is a crucial mediator in Alzheimer disease and amyotrophic lateral sclerosis.
- CyPA secretion increases during pro-inflammatory diseases, such as rheumatoid arthritis, sepsis and asthma.
- CyPA expression increases with aging.

## Open Questions

- Deepen the mechanisms by which CyPA contributes to the development of human diseases.
- Identify novel CyPA receptors able to explain all of the cellular events associated with CyPA.
- Design therapeutic agents with the capacity to block specific functions of CyPA while leaving other functions unaffected and without any effect on other CyP isoforms.

Cyclophilins (CyPs) are a family of ubiquitous proteins evolutionarily well conserved and present in all prokaryotes and eukaryotes.^1^ They have peptidyl prolyl isomerase activity, which catalyzes the isomerization of peptide bonds from *trans* form to *cis* form at proline residues and facilitates protein folding.^2^ Human CyPs consist of 16 family members that are structurally distinct. Among them, the most abundant member is CyPA, which makes up ∼0.1–0.6% of the total cytosolic proteins.^1, 3^ CyPA was initially purified from bovine thymocytes and identified as the primary cytosolic binding protein of the immunosuppressive drug cyclosporin A (CsA).^4^

CyPA is believed to have important roles in many biological conditions including protein folding, trafficking, and T-cell activation (Table 1).

Although CyPA is present intracellularly, it can be secreted from cells in response to inflammatory stimuli such as hypoxia, infection, and oxidative stress.^21, 24, 25, 26^

The secreted form of CyPA may mediate intercellular communication acting as an autocrine/paracrine factor. In fact, it was shown that extracellular CyPA stimulates pro-inflammatory signals in endothelial cells (EC) and vascular smooth muscle cells (VSMC),^21, 26^ and it is a nonmitogenic survival-enhancing autocrine factor that contributes to mouse embryonic stem cell growth.^27^ Further, extracellular CyPA has a potent chemotactic effect on leukocytes, monocytes, and lymphocytes.^25, 28^ The chemotactic activity of CyPA is mediated, in part, through the activation of the ubiquitous Ig like CD147 cell receptor.^29^

A growing body of evidence suggested its involvement in key processes underling human pathologies. The objective of this article is to review the current knowledge of CyPA regarding its potential role in several human diseases in order to offer novel therapeutic strategies.

## CyPA and Cardiovascular Disease

### Vascular remodeling

It has become clear that increases in reactive oxygen species (ROS) and inflammation represent key pathogenic mechanisms for vascular disease. For example, ROS have been implicated in the pathogenesis of neointima formation in part by promoting VSMC growth as well as stimulating pro-inflammatory events.^30^ In light of the major importance of CyPA in those aspect closely related to vascular remodeling, we analyzed the response to complete carotid ligation in wild-type (WT) mice, CyPA knockout (CyPA^−/−^) mice, and mice overexpressing CyPA specifically in VSMC (termed VSMC-Tg).^22^ Analysis of ligated arteries revealed that the intimal area as well as medial and adventitial thickening were significantly lower in CyPA^−/−^ mice and significantly enhanced in VSMC-Tg mice *versus* WT mice. Interestingly, there was a significant decrease in the recruitment of inflammatory cells in ligated arteries of CyPA^−/−^ mice and a significant increase in VSMC-Tg mice (approximately twofold) compared with WT mice. In addition, extracellular signal-regulated kinase 1/2 (ERK1/2) activation and Ki67^+^ cells were consistently decreased in CyPA^−/−^ mice after carotid ligation. These data strengthen the link between CyPA expression and VSMC proliferation and suggest that VSMC-derived CyPA is important for the recruitment of inflammatory cells.

### Abdominal aortic aneurysm (AAA)

**Figure 1:**
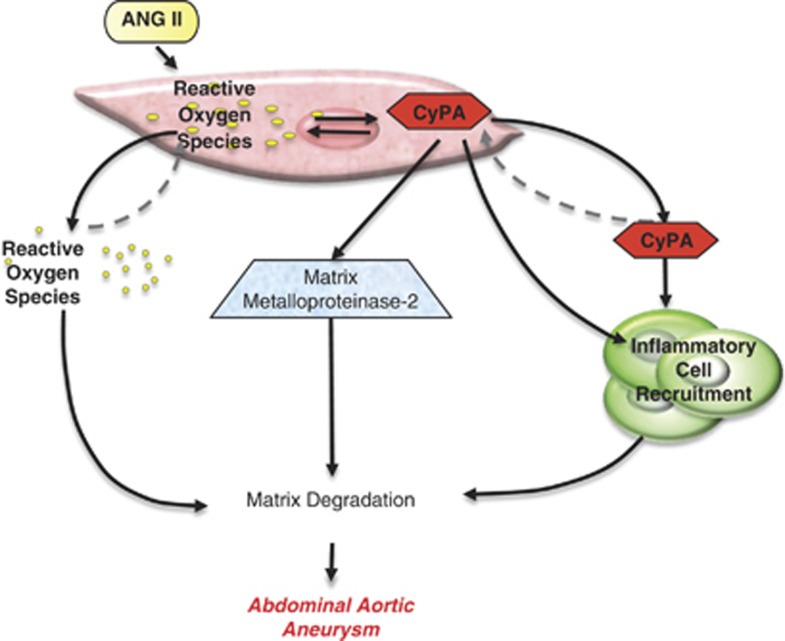
CyPA is involved in AAA formation. Ang II induces ROS formation; this in turn determines the release of CyPA from VSMC. Extracellular CyPA contributes to the recruitment of inflammatory cells in the aneurysm site and the activation of MMPs that degrade the extracellular matrix. CyPA itself induces ROS formation by a positive feedback loop

Inflammation has long been known to contribute to the pathogenesis of AAA. More than a decade ago, it was shown that treatment with CsA attenuated the formation of AAA in the rat model of elastase infusion.^31^ The authors of that study pointed out that the immunosuppressive effects of CsA would probably preclude its use in patients with aneurysms. We have recently demonstrated that deletion of CyPA in mice prevents the formation of AAA in response to infusion of angiotensin II (Ang II).^32^ Importantly, mice lacking CyPA were completely protected against aortic rupture leading to sudden death. Mechanistic studies revealed that deletion of CyPA reduces aortic inflammation, oxidative stress, and matrix degradation (Figure 1). In a series of experiments involving bone marrow (BM) transplantation, as well as experiments in which CyPA was selectively overexpressed in VSMC, we showed that expression of CyPA in VSMC, rather than BM–derived cells, is crucial to the development of AAA. Finally, to demonstrate its relevance to human aneurysmal disease, we showed that Ang II caused the release of CyPA and the activation of metalloproteinase 2 (MMP-2) in VSMC derived from human AAA.

Recently, Prins *et al.*^33^ found that benzo[a]pyrene potentiates the pathogenesis of AAA by increasing CyPA expression. Furthermore, simvastatin-treated patients with AAA exhibited lower CyPA mRNA, as well as CyPA intracellular protein levels.^34^ Thus, the interference with signaling pathways leading to CyPA may reveal a new strategy in the treatment of AAA.

### Atherosclerosis

**Figure 2:**
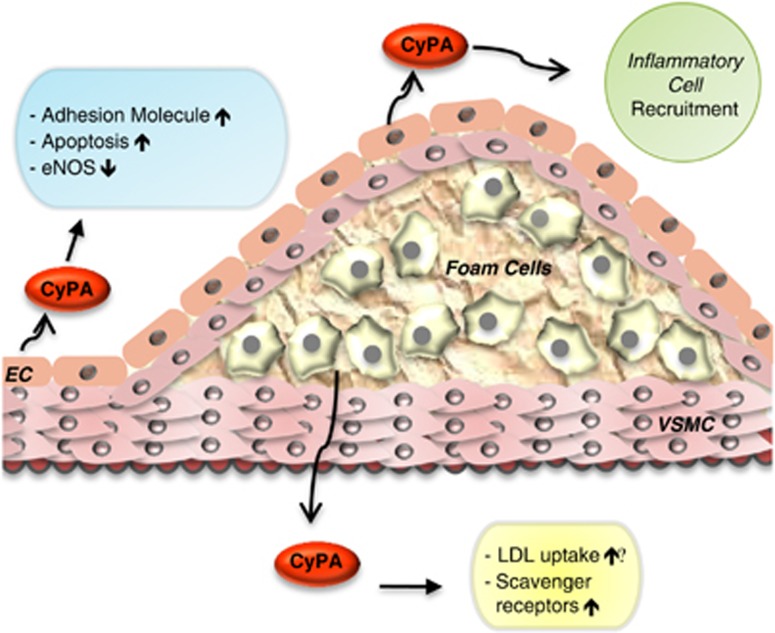
CyPA contributes to atherosclerosis formation. As atherosclerosis progresses, inflammation and ROS induce the release of CyPA from VSMC, EC and macrophages. Intracellular CyPA, as well as extracellular CyPA, influence LDL uptake by regulating the expression of scavenger receptors on the vessel wall. Also, CyPA induces inflammation of EC by increasing adhesion molecule expression and decreasing eNOS expression. In addition, CyPA increases the apoptosis of EC. Finally, CyPA induces the recruitment of inflammatory cells in atherosclerotic lesions

One of the major causes of AAA is atherosclerosis, a disease characterized by chronic inflammation of the arterial wall.^35^ Based on the premise that atherosclerosis stimulates AAA, we hypothesized that CyPA would necessarily have a significant impact on atherosclerosis development. We have characterized five important pathological mechanisms by which CyPA promotes atherosclerosis (Figure 2).^36^ First, CyPA stimulates low-density lipoprotein (LDL) uptake in the vessel wall by regulating the expression of scavenger receptors. Second, CyPA increases EC activation and inflammation by increasing vascular cell adhesion molecule 1 (VCAM-1) expression. Third, CyPA decreases endothelial nitric oxide synthase (eNOS) expression through kruppel-like factor 2 (KLF2) transcriptional repression in EC. Fourth, CyPA is a key determinant for tumor necrosis factor alpha (TNF-*α*)-induced EC apoptosis. Finally, CyPA stimulates recruitment of inflammatory cells derived from the BM to the aortic wall.

Thus, CyPA may have an important role in several stages of atherosclerosis. This observation is also supported by recent papers suggesting that CyPA is involved in the early phase of atherosclerosis by regulating fatty streak formation,^37^ and later phases by affecting plaque rupture^32, 38^ and thrombosis that complicate the disease.^39, 40^

**Figure 3:**
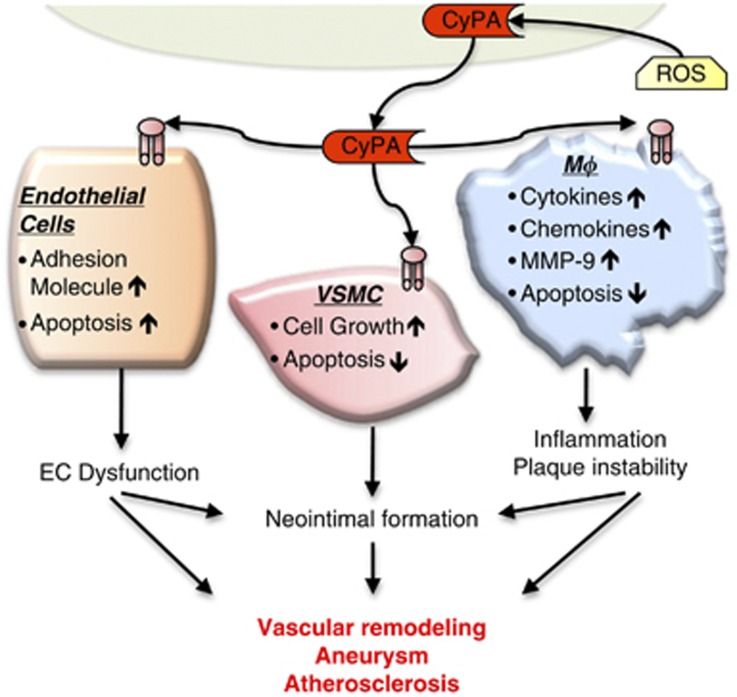
CyPA effects on EC, VSMC and macrophages. CyPA is secreted in response to ROS. Extracellular CyPA increases DNA synthesis and inhibits apoptosis in VSMC. In contrast, CyPA stimulates EC apoptosis, stimulates the expression of adhesion molecules including E-selectin and VCAM-1. Also, extracellular CyPA induces a pro-inflammatory status in macrophages by stimulating cytokines and chemokines production as well as inducing the activation of MMP-9 and decreasing apoptosis. These effects induce inflammation, EC dysfunction, neointimal formation thereby accelerating vascular remodeling, AAA and atherosclerosis

Based on the literature reported above, it is now clear that CyPA is a crucial mediator of vascular remodeling, AAA, and atherosclerosis, three diseases that share common pathways (Figure 3).

### Cardiac diseases

Inflammation triggered by oxidative stress is the cause of many human cardiac diseases including hypertrophy, myocardial ischemia, and coronary artery disease.^41^ Considering the role of CyPA in inflammation and ROS generation, we studied CyPA functions in heart disease. In particular, we evaluated the role of CyPA in Ang II–induced cardiac hypertrophy.^42^ Interestingly, mice lacking CyPA exhibited significantly less Ang II–induced cardiac hypertrophy. Most importantly, we proved that CyPA is required for Ang II–mediated cardiac hypertrophy by directly potentiating ROS production, stimulating the proliferation and migration of cardiac fibroblasts, and promoting cardiac myocyte hypertrophy.

In addition, May and coworkers^43^ demonstrated that CyPA is involved in myocardial ischemia and reperfusion injury.

Emerging data suggested that CyPA might be a valuable marker for predicting the severity of acute coronary syndromes. Yan *et al.*^44^ reported that serum CyPA concentration in unstable angina and acute myocardial infarction subjects are significantly higher than those in patients with stable angina and controls.

This study was confirmed by Satoh *et al.*^45^ that found that plasma levels of CyPA correlated with the anatomical severity of stable coronary artery disease.

In summary, CyPA is a critical determinant of cardiac hypertrophy, myocardial ischemia, and reperfusion injury and may be a helpful biomarker of coronary artery disease.

## CyPA and Diabetes

Recently, Ramachandran *et al.*^46^ presented evidence for a significant role of CyPA in the pathogenesis of diabetes. They performed a proteomic analysis of high glucose-primed monocytes, and identified CyPA as a potential secretory marker of inflammation in type 2 diabetes. Specifically, they reported that CyPA expression is reduced in circulating monocytes from patients with type 2 diabetes. In addition, they found that the levels of CyPA in plasma samples of patients with diabetes and coronary artery disease are higher in comparison with plasma obtained from healthy volunteers. These data suggested that CyPA secreted from monocytes could be an important pro-inflammatory stimulus for vascular inflammation in patients with diabetes.

## CyPA and Viral Infection

### CyPA and human immunodeficiency virus (HIV-1)

CyPA has been extensively studied from the gene to protein level during HIV-1 infection. CyPA is encoded by the peptidyl prolyl isomerase A (*PPIA*) gene, and regulatory *PPIA* polymorphisms were found to be a component of genetic susceptibility to HIV-1 infection or disease progression.^47^ In addition, analysis of sera from HIV-1-infected individuals has revealed higher protein concentrations of CyPA (about fourfold) compared with sera of healthy controls.^48^ Interestingly, HIV-1 replication was decreased in human CD4^+^ T cells when CyPA was knocked out.^49^ Molecular insights have revealed that CyPA can interact with HIV capsid proteins (CA domain).^5^ Furthermore, CyPA interacts with HIV accessory proteins, such as the viral protein R (Vpr) and Nef to facilitate a step in the viral life cycle between penetration and reverse transcription.^50, 51^ Recently, it has been reported that CyPA in dendritic cells could recognize the newly synthesized HIV-1 CA domain and subsequently prompt an interferon type I (IFN-I) response through activation of IRF3.^52^ Therefore, packaging of host CyPA into HIV particles is an important step in HIV morphogenesis and essential for HIV replication.

### CyPA and hepatitis virus

Similar to HIV-1, several lines of evidence indicate that CyPA positively regulates the replication of hepatitis C virus (HCV).^53^ Knockdown of endogenous CyPA significantly hampered HCV RNA replication and viral protein expression.^54^ Molecular studies provided evidence that CyPA enhances the replication of HCV by binding to the non-structural 5A (NS5A) and NS5B protein of HCV.^55^

CyPA is also important for hepatitis B virus (HBV) infection. Higher serum CyPA levels were detected in chronic hepatitis B patients than in healthy individuals.^56^

Studies on molecular mechanisms regulating HBV replication showed that CyPA interacts with the small surface proteins (SHBs) of the HBV surface antigen (HBsAg).^56^ It was hypothesized that CyPA binds to SHBs and is secreted along with HBsAg particles.^53^ Thus, CyPA may function as a cell-intrinsic sensor able to recognize HCV and HBV proteins in order to promote viral infection and replication.

### CyPA and influenza A virus

CyPA was found in the core of the influenza virion^57^ and was upregulated upon infection by avian H9N2 influenza virus in AGS cells (a human gastric carcinoma cell line).^58^ Liu *et al.*^59^ revealed that overexpression of CyPA blocked influenza A virus replication, whereas the depletion of endogenous CyPA resulted in enhanced production of influenza A virus. Furthermore, CyPA was found to interact with the matrix protein 1 (M1) affecting the early stage of viral replication. Finally, CyPA was able to restrict influenza virus replication through accelerating degradation of the M1 protein.

### CyPA and other viruses

It was reported that vaccinia virus (VV) infection led to an impressive increase in CyPA stability and CyPA is incorporated into the virus particle during morphogenesis.^60^ In addition, CyPA was found to act as a chaperone for the nucleocapsid protein of the vesicular stomatitis virus (VSV).^61^

CyPA was also reported to regulate severe acute respiratory syndrome coronavirus (SARS-CoV) replication through binding to the nucleocapsid protein and incorporation into particles.^62^

Then, it was shown that CyPA is required for the host IFN-I response in rotavirus (RV) infection of MA104 cells.^63^ Finally, Keyes *et al.*^64^ demonstrated that CyPA expression is an important factor in human cytomegalovirus (HCMV) infection and reactivation.

In light of the major importance of CyPA in the regulation of infectivity and replication of several viruses, it is worth proposing CyPA as target for anti-viral therapy.

## Protozoan Parasites

An emerging body of literature is reporting CyPA importance in the growth of protozoan parasites affecting humans. It has been suggested that the mammalian host cell CyPA seems to be involved in the intracellular replication cycle of *Leishmania major* parasites, as CyPA siRNA interference or CsA reduced the parasite burden.^65^ Some of the small CyPs from *Plasmodium falciparum* and *Toxoplasma gondii* (i.e. TgCyP18) have shown similar inhibitory profiles as those established for the human CyPA.^66, 67^

The involvement of CyPA in protozoan parasite growth was also supported by the fact that CsA has anti-parasitic activity against a wide variety of parasites^68^ with the exception of *Leishmania*.^69^

## CyPA and Cancer

### Upregulation of CyPA

There is now an established body of knowledge about the role of CyPA in cancer. Indeed, various reports have shown that CyPA is upregulated in cancer and is a key determinant for malignant transformation and metastasis.^70, 71^

In small cell lung cancer, overexpressed CyPA stimulates cancer cell growth, whereas CyPA knockdown slows down cancer cell growth.^70, 72^

CyPA is involved in diverse pathological processes of cancer development. Specifically, it has been reported that overexpressed CyPA in many cancers: (1) helps cancer proliferation,^73^ (2) regulates cell cycle progression,^74^ (3) blocks apoptosis,^75^ and (4) promotes cell migration/invasion.^76^

Interestingly, CyPA expression is influenced by chemotherapeutic agents. For example, treatment with anti-cancer drugs such as 5-aza-2-deoxycytidine, celecoxib, and 5-fluorouracil, decreases CyPA expression in cancer cells.^77, 78, 79^ Moreover, CsA and sanglifehrin A (SfA), the two immunosuppressive drugs that bind CyPA, increase the chemotherapeutic effect of cisplatin in glioblastoma multiforme.^80^

### Transcriptional regulation of CyPA

**Figure 4:**
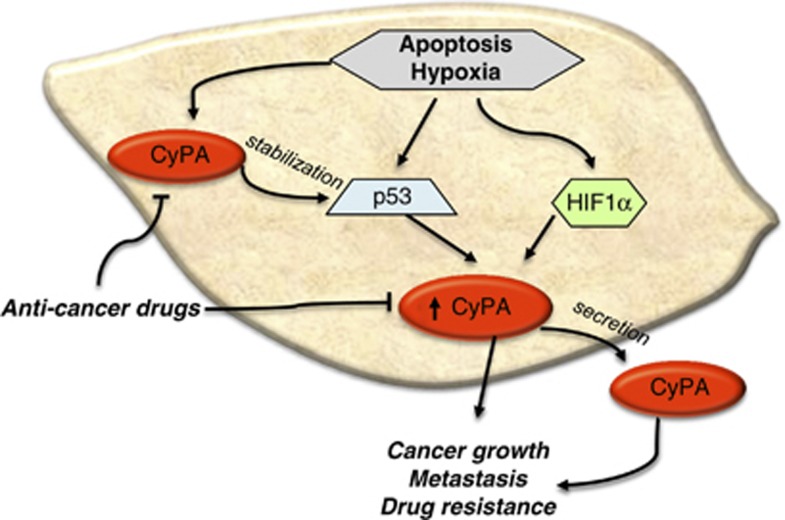
CyPA induces cancer growth. Apoptosis and hypoxia, through the activation of p53 and HIF1*α*, upregulate CyPA expression. This in turn stabilizes p53 protein. CyPA is also secreted by cancer cells and promotes cancer growth, metastasis and drug resistance. Anti-cancer drugs, such as DAC and 5-FU, decrease CyPA expression and thus block cancer progression. However, CyPA upregulation also decreases cisplatin-induced cell death suggesting that CyPA may lead to drug resistance

Recently, upregulation of CyPA in cancers was reported to be controlled by p53 and HIF1*α* (Figure 4), two critical transcription factors for cancer development. It was shown that CyPA protein is upregulated by p53-induced apoptotic conditions and CyPA may also confer p53 stability through its PPIase activity.^81^ However, overexpressed CyPA inhibits hypoxia- and cisplatin-induced cell death in a p53- independent manner. Furthermore, it was found that, under hypoxic conditions, CyPA, whose promoter contains the hypoxia response element (HRE) motif, can be induced by HIF1*α*.^75^ Thus, the regulatory relations of CyPA with p53 and HIF1*α* may present a new insight in understanding CyPA function during cancer development.

### Drug resistance

Choi *et al.*^75^ also found that CyPA overexpression decreases cisplatin-induced cell death, whereas CyPA knockdown lower cell survival rates. These results suggested that CyPA overexpression may lead to drug resistance, which results in a bad outcome of chemotherapy. Subsequent oligo-microarray analysis by Yu and collaborators^82^ revealed that CyPA can upregulate the expression of many cytokine and drug resistance-related genes including drug metabolism and drug transport-related genes. Some of these genes, such as IL-6, multidrug resistance-associated protein 2 (MRP2), MRP3, microsomal glutathione *S*-transferase 1 (MGST1), and glutathione transferase zeta 1 (GSTZ1), may contribute to drug resistance.^83^ In addition, CyPA promoted the expression of many ATP-binding cassette (ABC) transporters,^82^ which are able to reduce cellular drug accumulation and consequently induce drug resistance. It was also observed that liver cells stably expressing CyPA (SK-Hep1-CyPA) show an increased resistance for the anti-cancer drugs doxorubicin and vincristine. Also, the accumulation of doxorubicin was reduced in SK-Hep1-CyPA cells. These data suggest that the elevated expression of CyPA may contribute to clinical resistance to chemotherapy.

### *In vivo* studies

A report on CyPA in non-small-cell lung cancer revealed that CyPA knockdown has a significant effect on tumor growth *in vivo*.^72^ Two non-small-cell lung cancer cell lines, 5M2 and LC-103H, stably transfected with pSUPER-CyPA RNAi were grown as xenografts in severe combined immunodeficient (SCID) mice, and CyPA knockdown cells yielded slower-growing tumors than the cells transfected with scrambled RNAi. This effect was confirmed by overexpressing CyPA in the small airway epithelial cell line, S1LEK3. These cells resulted in faster growing xenografts in SCID mice compared with cells transfected with the empty vector. Large tumors were observed in the CyPA-overexpressing group, whereas none was observed in the vector group.^72^

Other interesting data by Li *et al.*^73^ revealed that intra-tumor injection of Pgenesil-2-CypA-shRNA decreases tumor development in nude mice. These data indicated that CyPA suppression by shRNA could significantly decrease tumor growth *in vivo*, with no apparent toxicity at the dose used.

## CyPA and Nervous System Degeneration

### Alzheimer disease

**Figure 5:**
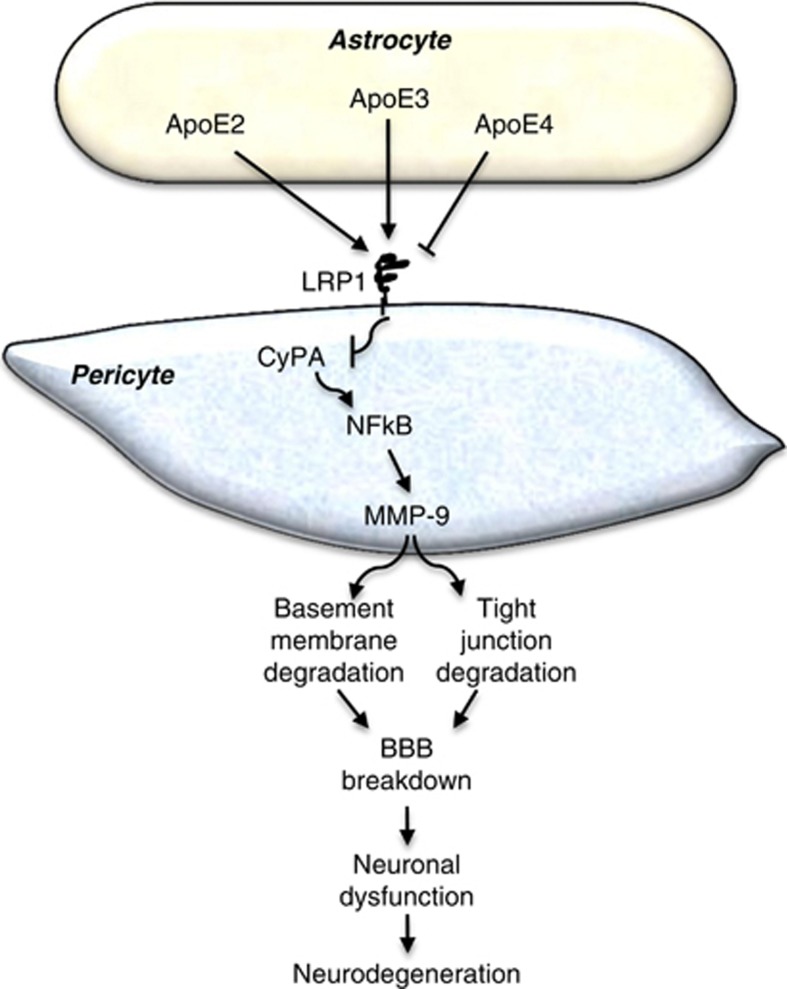
CyPA promotes neurodegeneration. ApoE2 and ApoE3 secreted by astrocytes, but not ApoE4, stimulates the low-density lipoprotein receptor-related protein 1 (LRP1) on pericytes. This event suppresses the pro-inflammatory pathway orchestrated by CyPA that leads to NF-kB and MMP-9 activation. As a consequence, the MMP-9-mediated degradation of tight junction and basement membrane proteins that causes BBB breakdown and neuronal dysfunction is inhibited

A growing body of evidence suggests oxidative stress involvement in neurodegenerative diseases.^84^ In addition, many papers showed the contribution of CyPA in oxidative stress mediated-diseases.^21, 30, 32^ Recently Bell *et al.*^85^ reported the involvement of CyPA in Alzheimer disease. In particular, they showed that astrocyte-derived human ApoE4 leads to an age-dependent progressive blood–brain barrier (BBB) breakdown (Figure 5). This event is driven by CyPA that initiates a pro-inflammatory pathway activating nuclear factor kappa B (NF-*κ*B) and MMP-9. The activation of this pro-inflammatory pathway in brain capillary pericytes determines the release of neurotoxic molecules from the vessels, damaging neurons and affecting their synaptic connections.

Another recent paper by Kayenda *et al.*^86^ illustrated that the CD147 receptor, by interacting with CyPA, can influence amyloid-*β* peptide levels, a protein that is central to Alzheimer's disease pathogenesis. Furthermore, it has been shown that copper affects the secretion of CyPA.^87^ This mechanism is of particular relevance in the nervous system regulation as copper dyshomeostasis is responsible for the neurological symptoms observed in genetically inherited copper-dependent disorders (i.e., Menkes' and Wilson's diseases), as well as Alzheimer disease.

### Amyotrophic lateral sclerosis (ALS)

It has been reported that mutations in Cu/Zn superoxide dismutase-1 (SOD1) can cause familial ALS.^88^ SOD1 transgenic mice that have the mutant human SOD1 (G93A) substitution are used as a model for ALS. Proteomic analysis of spinal cord of the G93A SOD1 mouse showed a variation in the isoform pattern of CyPA.^89^ In addition, overexpressed WT CyPA, but not CyPA with a rotamase active site point mutation, protected cells expressing ALS-related mutant SOD from death.^90^ Mechanistic insights revealed that CyPA is involved in motor neuronal cell death in ALS model mice by interacting with apoptosis-inducing factor (AIF).^14^ In particular, in the spinal cords of G93A SOD1 mice, the expressions of CyPA and AIF were detected in the motor neurons, and AIF co-translocated to the motor neuronal nuclei with CyPA. Furthermore, Basso *et al.*^91^ found that CyPA is significantly enriched in the insoluble fraction of spinal cords of ALS patients, thus may contribute to protein aggregate formation in ALS. Finally, Nardo *et al.*^92^ identified CyPA as biomarker for ALS in peripheral blood mononuclear cells and find an association with disease progression.

## CyPA and Rheumatoid Arthritis (RA)

Of interest, RA has been the first condition in which a secreted form of CyPA has been demonstrated in extracellular fluids. As shown by Billich *et al.*^93^, CyPA was increased in the synovial fluids of RA patients compared with knee osteoarthritis patients. Won-Ha Lee and his colleagues^94^ extended the findings on CyPA in RA by demonstrating that macrophages of the synovial lining layer constitute the major source of CyPA. In addition, they showed that stimulation of monocytes with CyPA results in increased production of inflammatory cytokines. Yang *et al.*^95^ also suggested that in RA, the CyPA-CD147 interaction might contribute to the destruction of cartilage and bone by upregulating MMP-9 expression and adhesion of monocytes/macrophages to extracellular matrix. Similarly, Wang *et al.*^96^ suggested that CyPA significantly enhances the secretion of MMP-2 and MMP-9, cell invasion, and inflammatory cytokines production of monocytes.

Recently, it has been demonstrated that the increased expression of CD147 on neutrophils in RA may be responsible for CyPA-mediated neutrophil migration into the joints, elevated MMPs secretion, and cell invasion of synoviocytes.^97^

Further, it was reported that CyPA affects IL-8-directed chemotaxis in neutrophils of RA patients.^98^

Altogether, these data are of interest because they tighten the links between CyPA and key pathological mechanisms of RA.

## CyPA and Sepsis

After the discovery of CyPA as an extracellular mediator in RA, the role of CyPA in other inflammatory diseases was evaluated. In 1997, Tegeder *et al.*^99^ reported that CyPA PPIase activity is significantly higher in patients with severe sepsis than healthy subjects. In addition, elevated PPIase activity was associated with high mortality. Then, Dear *et al.,*^100^ by using a mouse model of sepsis based on cecal ligation and puncture, found that CyPA increased in abundance in the liver after sepsis. Moreover, Huang and collaborators^101^ reported that CyPA expression is modulated in peripheral lymphocytes from *Pseudomonas aeruginosa*-induced sepsis^101, 102^ and scalded rabbits caused by *Staphylococcus aureus* invasion.^103^

Finally, a comparative proteome analysis was performed on protein extracts from human monocyte THP-1 cells stimulated with either Lipoteichoic acid (LTA) from *Staphylococcus aureus* (aLTA) and from *Lactobacillus plantarum* LTA (pLTA). Interestingly, CyPA expression was significantly changed by aLTA- or pLTA stimulation.^104^

It is concluded that CyPA is a mediator in the pathophysiology of sepsis and might acquire a diagnostic value.

## CyPA and Asthma

Constant and collaborators,^105^ using a mouse model of asthmatic inflammation, showed a correlation between CyPA and asthma. In particular, they found that (1) extracellular CyPA is elevated in the airways of asthmatic mice; (2) CyPA induces CD147-dependent chemotaxis of activated CD4^+^ T cells *in vitro*; (3) *in vivo* treatment with anti-CD147 mAb significantly reduces the accumulation of inflammatory cells in lung tissues; and (4) anti-CD147 treatment significantly reduces airway epithelial mucin production and bronchial hyperactivity to methacholine challenge.

Recently, Stemmy *et al.,*^106^ by using a new murine model of chronic allergic asthma, found elevated concentrations of extracellular CyPA, but not classic chemokines, in the chronic phase of asthma. Also, blocking the activity of CyPA reduced the recruitment and persistence of leukocytes. Interestingly, it was reported that CyPA is present in nasal wash samples of asthmatic patients during the chronic phase of the disease and its levels associate with clinical parameters of disease severity.^107^

## CyPA and Periodontitis

Recently Li *et al.*^108^ addressed the possible association of CyPA with pathological inflammation and destruction of periodontal tissues. They found that CyPA expression was dramatically elevated in inflamed gingival tissues as compared with healthy gingival tissues and co-localized in infiltrating macrophages and lymphocytes, as well as osteoclasts and osteoblasts in interradicular bone. As reported for other inflammatory diseases, CyPA expression correlated with MMP-1, MMP-2, and MMP-9 expression in inflamed gingiva. The same authors also collected gingival tissues and peripheral blood from patients with moderate to severe periodontitis or from healthy donors.^109^ CyPA was found to localize in the infiltrating cells and/or in the extracellular matrix in the inflamed gingival connective tissues. Also, CyPA was involved in the inflammatory response of periodontal tissues through inducing the chemotaxis of PBMCs/neutrophils and the secretion of TNF*α*/IL-8.

These data suggest that CyPA is associated with the inflammatory infiltration and alveolar bone destruction of periodontitis.

## CyPA and Aging

Another interesting aspect of CyPA functions is its correlation with aging. Recently, Li *et al.*^110^ showed that the expression of CyPA increased with aging in normal skin, regardless of whether it was exposed or shaded. Similarly, it has been discovered that the expression level of CyPA is upregulated in the epidermis of aged people compared with younger people.^111^ Further, CyPA expression was found significantly higher in old rats than in the younger ones.^112^ In contrast, proteomic analysis of dermal fibroblasts cultured *in vitro* from human healthy subjects of different ages revealed that CyPA was markedly reduced with age, consistent with the reduced *de novo* synthesis of proteins and failure in performing biologically active conformational changes frequently observed during aging.^113^

## CyPA Inhibitors and Their Pharmacological Use

As outlined above, CsA is a CyPA inhibitor and is the most well studied and tightest binding CyPA ligand identified to date.^4^ CsA binds both extracellular and intracellular CyPA and inhibits its PPIase activity. In particular, it inhibits the protein phosphatase calcineurin and blocks the translocation of nuclear factor from activated T cells (NF-AT) from the cytosol to the nucleus, thus preventing the transcription of genes encoding pro-inflammatory cytokines.^114, 115^

Yurchenko *et al.*^29^ reported that CD147 interacts with extracellular CyPA in a CsA-sensitive fashion. Others showed that CsA blocks the folding of some proteins^9, 10^ and modulates protein trafficking in cells.^16, 116, 117^

More importantly, CsA was shown by Borel *et al.*^118^ to be a potent and specific inhibitor of T-cell responses to alloantigens. This discovery led to widespread clinical use of CsA as an immunosuppressant, revolutionizing organ transplantation in humans. However, multiple side effects were observed with the long-term use of CsA in organ transplant recipients, especially severe nephrotoxicity, which remains one of the major obstacles for a broader use of the drug in the treatment of inflammation and other immune disorders.^119^

Thus, compounds that selectively inhibit CyPA without immunosuppressive effects are urgently required and might be applicable to the therapy of human diseases. In this case, treatment with isoenzyme specific inhibitors (1) would probably elicit fewer side effects than a global CyP inhibitor like CsA and (2) would not get dispersed into a cellular sink when sequestered by other CyPs.

Recently, scientists have synthesized derivatives of CsA that lack calcineurin-binding properties and thus do not exhibit immunosuppressive effects. The anti-inflammatory effect of these compounds is achieved because they directly block the pro-inflammatory and chemotactic functions of extracellular CyPA.

A growing body of scientific evidence has indicated that these non-immunosuppressive analogs of CsA may have applications in multiple therapeutic areas. In particular, they are among the most promising of the new anti-HCV agents under development. For example, Alisporivir (Debio 025) is the first-in-class CyPA inhibitor that recently initiated a phase III trial. It binds to CyPA and shows additive anti-viral effect with pegylated interferon-alpha (pIFN-*α*) in patients with genotype 1 and 4 HCV.^120^

In addition, another derivative of CsA, NIM811, has been shown to promise anti-inflammatory effects in different experimental models associated with inflammatory response, such as acute lung injury and arthritis.^121, 122^

Another current challenge is to design therapeutic agents with the capacity to block specific functions of CyPA, while leaving other functions unaffected. In this context, a study reported the development of a small molecule with the capacity to specifically inhibit CyPA functions, without any effect on other CyP isoforms.^123^

Recently, a potent CyPA inhibitor, MM218, which can selectively inhibit the extracellular fraction of CyPA, has been synthesized.^124^ It appears to have a stronger anti-inflammatory effect than CsA, as well as no detectable adverse effects in an animal model of acute lung injury^125^ and myocardial reperfusion injury.^126^

It is hoped that the discovery of these novel selective drugs for CyPA might lead to a number of possible therapeutic applications in inflammatory disease.

## Conclusions and Perspectives

Based on current knowledge reviewed above, it is now clear that CyPA has multi-functional properties. A number of biological studies demonstrated that CyPA is involved in various cellular functions including protein folding, trafficking, assembly, immune-modulation, and cell signaling.^127^

In light of what is known about CyPA, it is believed that an increase in CyPA expression contributes to pathological conditions. In fact, CyPA^−/−^ mice are protected from vascular remodeling,^22^ aortic aneurysm,^32^ atherosclerosis,^36^ cardiac hypertrophy,^42^ and ischemia/reperfusion injury.^43^ In addition, CyPA knockdown decreases tumor growth *in vivo*^72, 73^ and CyPA inhibitors are the most promising of the new ant-HCV agents.^120^ Furthermore, several studies suggested that high CyPA expression correlates with poor outcome of patients with inflammatory diseases.^92, 107, 128^

It is important to underline that both the intracellular and extracellular form of CyPA are involved in its pathogenic actions. Specifically, we found that, under inflammatory conditions, CyPA is secreted from cells and acts as an autocrine and paracrine factor that exacerbates oxidative stress and inflammation.^22, 32, 42^ However, extracellular CyPA has been found to have a biphasic effect, by promoting cell proliferation and migration at low doses and cytotoxic effects at higher doses.^129^

The mechanism by which CyPA contributes to the development of inflammatory diseases may involve a complex interplay of proteins and pathways including the membrane receptor for CyPA, CD147. However, it is worth mentioning that a number of mechanistic details are still unknown and await further studies. Indeed, to date CD147 has not been proven unequivocally to mediate all of the cellular events associated with CyPA. Thus, the identification of novel receptors for CyPA would be essential for future translational efforts aimed at targeting the CyPA pathway.

In conclusion, it is believed that the development of agents that selectively inactivate CyPA or that block binding of CyPA to its receptor could prove to be a fruitful approach to forestalling several human diseases.

## Figures and Tables

**Table 1:** Major CyPA functions in protein folding and trafficking, T-cell activation and cell signaling

**CyPA functions**	**CyPA targets**	**CyPA effects**	**References**
Folding	HIV-1 Gag	Promotion of both the formation and the infectivity of virions of HIV-1	^5, 6^
	Homo-oligomeric a7 neuronal nicotinic receptor (nAChR) and type 3 serotonin receptor (5HT3R)	Maturation of the homo-oligomeric receptor	^7, 8^
	Collagen	Acceleration of the folding of the procollagen I	^9^
	Transferrin	Acceleration of an initial stage in transferrin folding	^10^
	*Drosophila rhodopsin*	Folding and stability of R1-6 rhodopsin by the NinaA-encoded protein (an eye-specific CyP)	^11, 12^
Trafficking	Apoptosis-inducing factor (AIF)	Co-translocation to the neuronal nuclei to induce cell death after cerebral hypoxia-ischemia and ALS	^13, 14^
	Heterogeneous nuclear ribonucleoprotein A2 (hnRNP A2)	CXCR4-mediated nuclear export of hnRNP A2, nuclear translocation of ERK1/2, and chemotactic cell migration	^15^
	CD147	Transport to the plasma membrane	^16^
	Asialoglycoprotein receptor (ASGPR)	Distribution of ASGPR between the plasma membrane and the endosomal pool	^17^
	Fructose-1,6-bisphosphatase (FBPase)	Import of FBPase into intermediate transport vesicles for vacuole delivery	^18^
	Zinc-finger protein 1 (Zpr1)	Promotion of nuclear export	^19^
T-cell activation	Interleukin-2 tyrosine kinase (Itk)	Positive regulation of Th1 profile and inhibition of Th2 differentiation	^20^
Cell Signaling	ERK1/2	Stimulation of VSMC growth	^21, 22^
	JNK, p38 kinase, and IkB, NF-kB, E-selectin, and VCAM-1	Stimulation EC apoptosis and inflammation	^23^

## Abbreviations

EC: endothelial cells
VSMC: vascular smooth muscle cells
ROS: Reactive Oxygen Species
ERK1/2: Extracellular signal-regulated kinase 1/2
Ang II: angiotensin II
BM: bone marrow
LDL: l*ow-density lipoprotein*
VCAM-1: vascular cell adhesion molecule 1
eNOS: endothelial nitric oxide synthase
KLF2: kruppel-like factor 2
TNF*α*: tumor necrosis factor alpha
Vpr: viral protein R
IFN-I: interferon type I
M1: matrix protein 1
HRE: hypoxia response element
MMP: metalloproteinase
NS5A: non-structural 5A
IL: interleukin
MRP: multidrug resistance-associated protein
MGST1: microsomal glutathione *S*-transferase 1
GSTZ1: glutathione transferase zeta 1
ABC: ATP-binding cassette
AIF: apoptosis-inducing factor
SOD1: superoxide dismutase-1
NF-*κ*B: nuclear factor kappa B
NF-AT: nuclear factor from activated T cells
pIFN-*α*: pegylated interferon-alpha
